# Genetic evidence for causal effects of immune dysfunction in psychiatric disorders: where are we?

**DOI:** 10.1038/s41398-024-02778-2

**Published:** 2024-01-26

**Authors:** Olena Iakunchykova, Esten H. Leonardsen, Yunpeng Wang

**Affiliations:** Lifespan Changes in Brain and Cognition (LCBC), Department of Psychology, University of Oslo, 0317 Oslo, Norway

**Keywords:** Genomics, Psychiatric disorders

## Abstract

The question of whether immune dysfunction contributes to risk of psychiatric disorders has long been a subject of interest. To assert this hypothesis a plethora of correlative evidence has been accumulated from the past decades; however, a variety of technical and practical obstacles impeded on a cause-effect interpretation of these data. With the advent of large-scale omics technology and advanced statistical models, particularly Mendelian randomization, new studies testing this old hypothesis are accruing. Here we synthesize these new findings from genomics and genetic causal inference studies on the role of immune dysfunction in major psychiatric disorders and reconcile these new data with pre-omics findings. By reconciling these evidences, we aim to identify key gaps and propose directions for future studies in the field.

## Introduction

Psychiatric disorders have surpassed physical disorders to become the leading causes of disability worldwide [1] but, still, our understanding of their underlying pathophysiology remains incomplete. One of the contemporary hypotheses is the “two-hits” or “multi-hits” model—in the context of genetic predispositions to these psychiatric disorders (the first hit; e.g., schizophrenia, SCZ), the accumulating effects of a second or more adverse exposures (the second hit) may lead to the diagnoses [2–4]. Perturbations to the immune system by these adverse exposures, such as infection [5–8] and stress [9–12], are an integral part of this hypothesis [2–4].

The immune system is the key machinery in protecting the body from exogenous and endogenous threats [13]. It, generally, comprises two interconnected arms, the innate and adaptive arm. The innate immunity is the first line of defense when the body faces threats: it responds swiftly and intensely but not specific to antigens. On the contrary, the adaptive arm responds by the coordination of a range of T cells and B cells that are targeted to the specific antigens in an elegant way, but may require days to fully take in action. Proper functioning of the immune system requires responses from both arms in concert, involving harmonized expression of a large network of molecules, e.g., the signaling molecules—cytokines, within a proper context [14]. In addition, the immune system is dynamic, and both its composition and responses change with development and aging [15]. Unfortunately, this complex nature of the immune system is predisposed to dysregulations which underlie many human diseases [16–19].

The relationship between immune dysfunction and psychiatric disorders have been extensively explored in the literature. Findings from epidemiological or clinical studies have recently been synthesized in several excellent meta-analyses and reviews [20–28]. Thus, we will not review them in depth here; instead, we will, after briefly summarizing the key findings from these studies, focus on recent evidence reported from large-scale genomic studies. Specifically, we highlight the use of Mendelian randomization (MR) [29] for causal inference in psychiatric disorders, which have not been thoroughly reviewed yet. Due to the requirement of large-scale data, MR studies to date have been performed on data shared by few consortia, and resulted in a significant data overlap, making it difficult to conduct a comprehensive meta-analysis. We will employ a narrative approach to summarize these findings and discuss their implications for future research.

### Epidemiological and clinical findings

The initial evidence for the involvement of immune dysfunction in psychiatric disorders came from studies examining the relationships between infections and psychiatric diagnosis [5, 7, 8, 30–33]. Maternal infections or exposures to pathogens have long been suggested as risk factors to psychiatric illness in offspring, for example, attention-deficit/hyperactivity disorders (ADHD) [34], autism spectrum disorder (ASD) [35], schizophrenia (SCZ) [5] and others [6, 33]. Postnatal early life infections have also been extensively reported to associate with later diagnosis of SCZ [5, 36], bipolar disorder (BIP) [31] and ASD [35]. As infection events preceded disorder diagnoses, these findings seemingly dictate potential causal relations. But their limitations are also apparent. Among these studies, there was considerable variability in pathogen types, i.e., from virus (e.g., cytomegalovirus), bacterium to parasite (e.g., toxoplasma gondii), in the infected body systems (e.g., respiratory, gastrointestinal), in the time of infection (the trimester or age of infected) and in severity of the infections examined. Therefore, these findings have not yet reached a consensus on the kind of infections that causes mental health problems later in life. In addition, other forms of confounding have not been carefully considered or controlled for. For example, a recent study has argued that the association of maternal infections during pregnancy with ADHD in offspring could be fully explained by the unmeasured familial confounding when applying a sibling comparison design [37]; another large-scale epidemiological study has reported that the diagnosis of SCZ was associated with increased risk of infection later in life, suggesting a causation in the opposite direction [38].

Chronic inflammation, as indexed by inflammatory marker levels, has been extensively studied as a potential risk factor to psychiatric disorders. Individuals diagnosed with these disorders frequently also develop immune and inflammation related conditions [39–41]. For instance, SCZ patients were reported having a 45% higher risk for developing autoimmune disorders compared to the general population. In addition, it has been shown that in most psychiatric patients the pro-inflammatory cytokine levels (e.g., interleukin 6 (IL6), IL1b, C-reactive protein (CRP)) were higher than in healthy controls [22, 23]. Prospective studies had indicated that elevated baseline levels of pro-inflammatory markers were predictive of psychiatric diagnoses many years later [20, 42–44]. In clinical settings, levels of these markers were also shown to decrease after treatment in patients [45]. There is evidence for efficacy of some agents with anti-inflammatory properties on first episode psychosis and schizophrenia [46], and reports of anti-inflammatory effects of anti-psychotic drugs are suggesting that their effectiveness is partially due to a modulating effect on the immune system [47]. Patients who respond to antidepressants have lower neuroinflammatory markers compared to non-respondents [48]. Selective and nonselective cytokine inhibitors like non-steroidal anti-inflammatory drugs were effective in improving depression in meta-analysis despite significant heterogeneity of individual studies [49]. A scoping review of anti-inflammatory medications for the treatment of mental disorders suggested different underlying mechanisms for their treatment success in BIP/MDD and SCZ [50].

Despite being promising, epidemiological findings are correlational in nature. The observed immune-psychiatric relationships can be interpreted in multiple non-exclusive ways: (1) Immune dysfunction leads to an increased risk of psychiatric disorders; (2) Immune dysfunction is the consequence of psychiatric disorders or medication for chronic illness; or, (3) unknown/unmeasured variables cause both—confounding effects. While prospective studies seemingly meet the Temporality criterion in Hill’s causal criteria [51], inflammatory markers were typically measured at baseline, thereby not fully capturing chronic immune process in a timely manner. Further, developing psychiatric disorders may take years, thus, even the compliance with this temporality assumption may be questioned.

### Genomic association studies’ findings

**Fig. 1:**
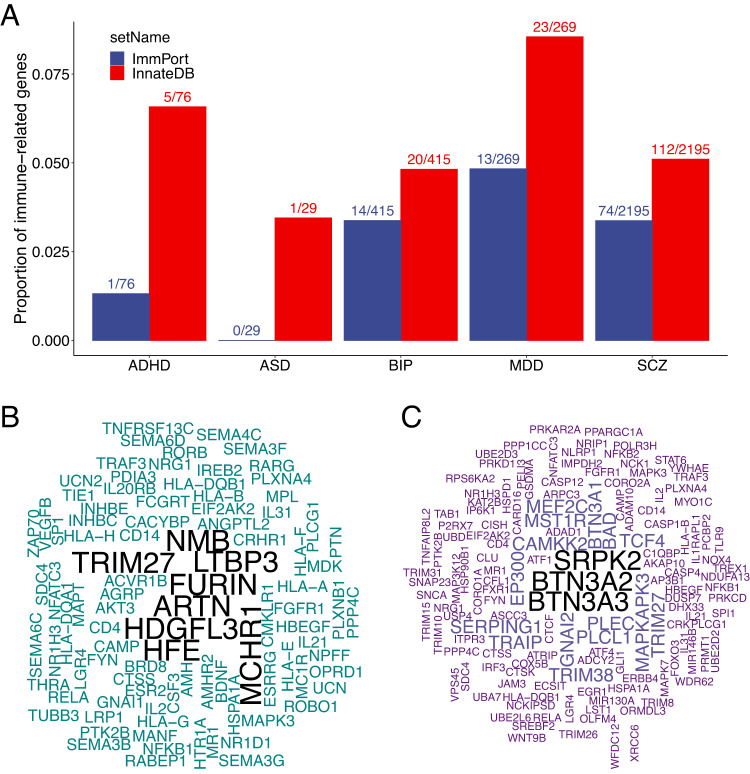
Psychiatric disorder associated genes identified by GWAS in relation to immune systems. **A** Genes reported by the psychiatric genomic consortium for attention-deficit/hyperactive disorder (ADHD), autism spectrum disorder (ASD), bipolar disorder (BIP), major depression disorder or depression (MDD) and schizophrenia (SCZ) are annotated to genes involved in innate immune response (InnateDB, https://www.innatedb.com/) and ImmPort (https://www.immport.org/). Proportions of reported genes annotated to each immune gene source are shown on *y* axis. The number of annotated genes and reported genes are shown on the top of each bar. WordCloud plots for reported genes annotated to ImmPort (**C**) and InnateDB (**B**). Text font sizes indicate the number of disorders associated with the gene.

In the last decade, genome-wide association studies (GWAS) have become a popular design in interrogating genetic associations with psychiatric disorders. In this design, millions of genetic variants are tested for association with a disorder without predefined biological hypotheses. Because germline variants are stable after conception, associations identified by this design are unaffected by reverse causation, a major problem in epidemiological studies. In the last 15 years, the psychiatric genomic consortium (PGC) has analyzed data from tens of thousands patients and healthy controls identifying a large number of genetic associations [52–56], for example, 287 loci for SCZ, 64 for BIP, 27 ADHD and more than 44 for major depression disorders (MDD), many of which were previously unknown. Within these identified loci, immune-related genes have been annotated (Fig. 1): for example, one of the first annotated immune gene is the HLA region gene for SCZ [57, 58].

Leveraging massive summary statistics from large-scale GWAS, polygenic genetic overlaps between immune-related and psychiatric disorders have been examined by genetic correlation estimates (gr) which quantifies the degrees of genetic sharing between traits. Significant positive genetic correlations between SCZ and Crohn’s disease (CD; gr = 0.097), inflammatory bowel disease (IBD; gr = 0.117), ulcerative colitis (UC; gr = 0.106), primary biliary cirrhosis (PBC; gr = 0.131) and psoriasis (PSO; gr = 0.182) have been reported [59–61]. Among these immune-related disorders, CD (gr = 0.22), UC (gr = 0.23), PSO (gr = 0.29), along with Celiac disease (gr = 0.34) were genetically correlated with BIP; rheumatoid arthritis (RA; gr = 0.16), type 1 diabetes (gr = −0.14), PSO (gr = 0.23) were genetically correlated with ADHD [61]. Moreover, in a recent study based on the UK Biobank (UKBB) data, CRP was shown genetically correlated with MDD (gr = 0.154), ADHD (gr = 0.326), obsessive-compulsive disorder (gr = −0.201), anorexia nervosa (gr = −0.268), post-traumatic stress disorder (gr = 0.238) and a trend (i.e., *p* > 0,05) was observed with SCZ (gr = −0.058) [62]. Another study reported genetic correlation of smaller magnitude (gr = 0.098) using summary GWAS from PGC (MDD) and CHARGE consortium (CRP) [63].

**Fig. 2:**
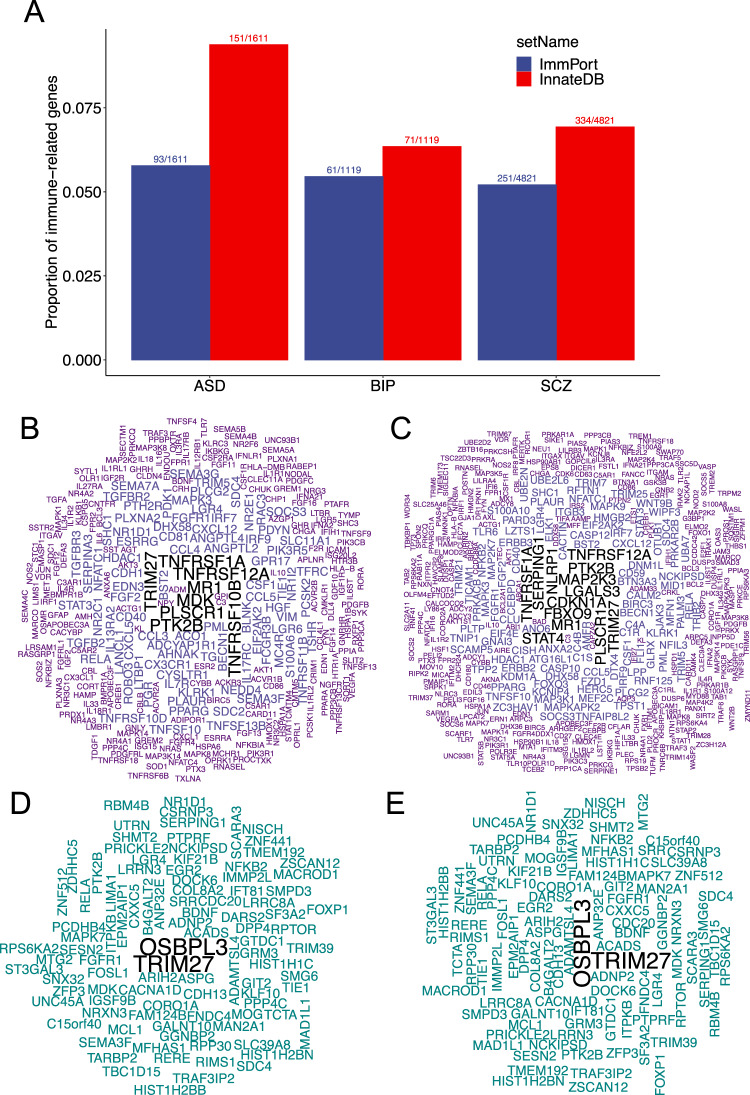
Psychiatric disorder associated genes identified by differential expression in brain tissue, GWAS, EWAS in relation to immune systems. **A** Genes identified by differential expression in brain tissue by Gandal et al. [66] for autism spectrum disorder (ASD), bipolar disorder (BIP), and schizophrenia (SCZ) are annotated to genes involved in innate immune response (InnateDB, https://www.innatedb.com/) and ImmPort (https://www.immport.org/). Proportions of reported genes annotated to each immune gene source are shown on *y* axis. The number of annotated genes and reported genes are shown on the top of each bar. WordCloud plots for reported genes annotated to ImmPort (**C**) and InnateDB (**B**). Wordcloud plots for reported genes identified by brain tissue expression, GWAS and EWAS annotated to ImmPort (**D**) and InnateDB (**E**). Text font sizes indicate the number of disorders associated with the gene.

To assess the immune-psychiatry relations at gene level, we extracted genes reported by the most recent PGC studies for the five disorders (SCZ, BIP, MDD, ADHD and ASD) with largest sample size so far. We compared these genes with those included in two curated immune databases: (1) the InnateDB [64] which includes genes involved in the innate immunity arm and (2) the ImmPort [65] which collects genes involved in general immune functioning (Fig. 1A). As anticipated, all the five disorders exhibit numerous genes associated with immune functioning. The WordClouds generated from the genes associated with these disorders and in the two immune databases highlight that both the innate and the adaptive immune arms were involved in risk of psychiatric disorders (Fig. 2B, C) and that several immune genes are associated with more than one disorder.

**Fig. 3:**
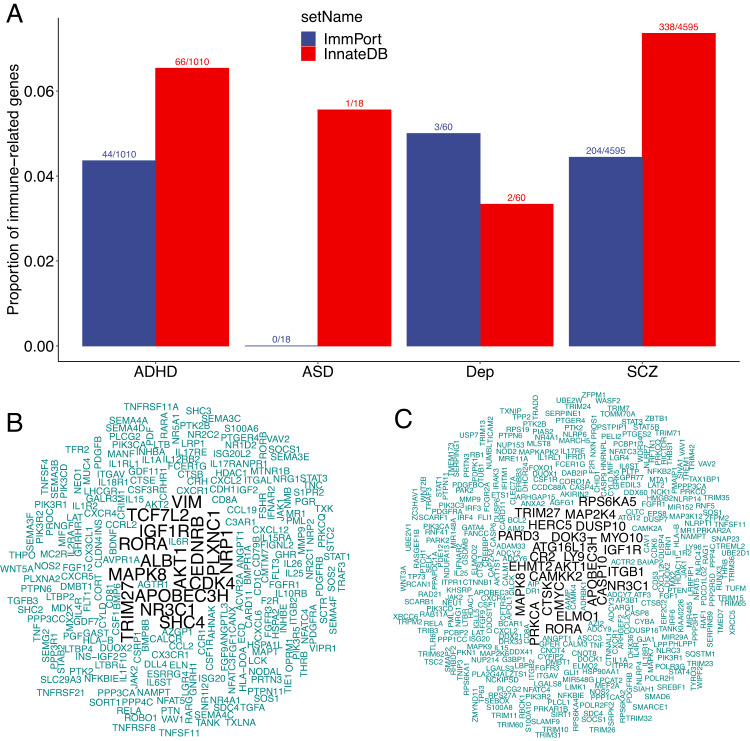
Psychiatric disorder associated genes identified by EWAS in relation to immune systems. **A** Genes identified by epigenomic wide association studies included in the EWAS Catalog (http://ewascatalog.org/; accessed at January 2023) for attention-deficiency/hyperactive disorder (ADHD), autism spectrum disorder (ASD), depression or depressive symptoms (Dep) and schizophrenia (SCZ) are annotated to genes involved in innate immune response (InnateDB, https://www.innatedb.com/) and ImmPort (https://www.immport.org/). Proportions of reported genes annotated to each immune gene source are shown on *y* axis. The number of annotated genes and reported genes are shown on the top of each bar. Wordcloud plots for reported genes annotated to ImmPort (**C**) and InnateDB (**B**). Text font sizes indicate the number of disorders associated with the gene.

Gene expressions vary in human tissues and are partially regulated by DNA methylations. We curated genes implicated by DNA methylation association studies (epigenome wide association studies (EWAS)) performed on blood samples from the EWAS Catalog and genes differentially expressed in brain tissues from psychiatric disorders and healthy controls reported by Gandal et al. [66]. We aligned these genes with those from the two immune gene databases noted above (Figs. 2 and 3). Similar to the observations from GWAS data, both the innate and adaptive immune systems were indicated (Figs. 2B, C and 3B, C). Together, 83 genes implicated in associations with SCZ were identified in the three types of studies; 16 genes for BIP by the two available studies, and two for ADHD (*FOXP1* and *ST3GAL3*). WordCloud plots created from these overlapping genes demonstrated that two immune genes, *OSBPL3* and *TRIM27*, were associated with both SCZ and BIP (Fig. 2D, E).

In sum, results from large-scale hypothesis-free genetic studies support the involvement of immune dysfunction in psychiatric disorders. The slightly divergent overlapping patterns among the three types of data sources may be due to differences in study sample sizes, biological nature of DNA variation, methylation and expression, where the latter two vary in age [67] and tissues. It is of note that measuring DNA methylation or gene expressions after diagnosis may represent a consequence of the disorders instead of a cause, and thus cause-effect interpretations for such studies need additional evidence. Another key observation is that, for the three types of studies, the number of genes involved in the overall immune system is not statistically enriched in psychiatric disorders: Assuming 20,000 genes in the genome [68] while there are 1376 and 1509 genes included in the two databases, on average 6–8% of identified psychiatric genes should be involved in the immune system at random; with this threshold, barely any psychiatric disorder would show statistically significant enrichment signal; but, this does not rule out that certain sub-immune response pathways, for example the NF-kb system [66] may be enriched.

### Mendelian randomization findings

Mendelian randomization (MR) analysis has been designed aiming to interrogate the cause-effect relations between exposures (here, immune functioning) and outcomes (here, psychiatric disorders) [29, 69]. In MR, genetic variants (e.g., SNPs) are used as proxies to the exposure of interest in a way to examine the causal effect of the exposures on outcomes. As per the Mendelian inheritance laws, genetic variations are randomly inherited from one generation to the next. MR design, under certain conditions such as random mating, can be regarded as a natural randomized trial. In addition, as germline genetic variants are stable, they are less likely affected by environmental factors during lifetime. Therefore, MR estimates are unbiased by reverse causation and confounders for exposures and outcomes (Box 1), in distinction to traditional observational studies. MR analysis can be performed either using the individual-level data (one-sample MR) or summary statistics from GWAS of two independent samples (two-sample MR), one for exposure and the other for outcome; with certain assumptions, the underlying models for these two designs are equivalent.

As publicly sharing of GWAS summary statistics is becoming the norm in the field, MR turns into the most cost-effective causal inference model for examining the relations between the immune dysfunction and psychiatric disorders. As such, the choices of exposures and outcomes in MR are intrinsically constrained by the data that is currently available. We have searched PubMed database for MR studies published in the past 10 years focusing on immune function and major psychiatric disorders (Box 2 and Table 1). In total, we have found 15 studies that investigated the relationships between inflammatory markers and psychiatric disorders (up to nine different diagnoses). Among these 15 studies, 13 used GWAS results from PGC, and for exposures, only a few common GWAS results were used (Table 1).

**Table 1:** Summary of findings from MR studies on determining causality between various markers of systemic inflammation and psychiatric disorders.

Psych disorders	Psych GWAS	Markers	Marker GWAS	MR models	Instrument selection	Findings (OR or *Beta*)	Study (year)
ADHD [91], AN [92], ASD [55], BIP [93], MDD [54], OCD [94], PTSD [95], SCZ [52], TS [96]	PGC	CRP, IL6, IL6R	Ligthart et al. [71]; Folkersen et al. [97]	2SMR; LCV; MVMR	GW-SNPs	CRP ⊣ SCZ (0.91)^a^ CRP ⊣ AN (0.91)^a^	Reay et al. (2022) [98]
ADHD [91], ASD [55], BIP [93], MDD [54], SCZ [73]	PGC	CRP	UKBB [99] and CHARGE-1000 Genomes [72]	2SMR	GW-SNPs	CRP ⊣ SCZ (−*0.11*)^b^ CRP ⊣ ASD (*−0.08*)^b^ CRP → ADHD (*0.1*)^b^	Koskeridis et al. (2022) [62]
ADHD [91], AN [100], ASD [55], BIP [93], MDD [101], OCD [94], SCZ [73]	PGC	41 inflammatory markers	Ahola-Olli et al. [102]	2SMR	GW-SNPs	BIP ⊣ IL9 (*−0.27*)^c^ MDD ⊣ SCGFb (*−0.58*)^c^ SCZ ⊣ CTACK (*−0.11*)^c^ FGFBasic ⊣ AN (0.4)^c^	Chen et al. (2022) [79]
Depressive and anxiety symptom score (DSS, ASS); GAD	UKBB [99]	CRP, IL6-signaling	UKBB [99]; Ligthart et al. [71]	1SMR; 2SMR	Cis-SNP	CRP ⊣ DSS (0.88)^d^ CRP ⊣ ASS (0.87)^d^ CRP ⊣ GAD (0.82)^d^ IL6 → DSS (1.34)^d^	Ye et al. (2021) [75]
SCZ [103], MDD [54], BIP [53]	PGC	IL6, TNFa, IL12, IL16, IL17, IL18, IL1RA, IL10, IL13, IL8, MCP1, sIL2Ra, IL4, IL7, IL9, IL5, CRP, Neutrophils, lymphocytes	The IL6R MR Consortium [104]; Sarwar et al. [105]; Ahola-Olli et al. [102]; Astle et al. [106]	2SMR; MVMR	GW-SNPs; cis SNPs	IL6 signaling → SCZ (1.24)^e^ IL6 → MDD (1.08)^e,h^	Perry et al. (2021) [80]
MDD [54, 101], Depressive symptom score	PGC; UKBB [99]	CRP; sIL6R	Ligthart et al. [71]; Sun et al. [107]	2SMR	Cis-SNPs	IL6 signaling → Suicidality (*0.01*)^c^	Kappelmann et al. (2021) [108]
Depressive and anxiety symptom score	UKBB [99]; NESDA [109]	CRP; IL6	UKBB [99]; NESDA [109]; Ligthart et al. [71]; The IL6R MR Consortium [104]; The IL6R Genetics Consortium Emerging Risk Factors Collaboration [105]	1SMR; 2SMR	Cis-SNPs	CRP ⊣ Suicidal ideation (*−0.24*)^b^ CRP ⊣ Cognitive problems (−*0.1*)^b^ CRP ⊣ psychological summary score (*−0.11*)^b^ IL6 fatigue (*0.25*)^f^ IL6 → Sleep problem (*0.19*)^f^	Milaneschi et al. (2021) [44]
MDD [54], recurrent depressive symptoms	PGC, UKBB [99]	sIL6R; CRP; sgp130	Van Dongen et al. [110]; Folkersen et al. [97]; Sun et al. [107]; Suhre et al. [111]; Yao et al. [112]	2SMR	GW-SNPs; Cis-SNPs	sIL6R → depression (1.02)^g^	Kelly et al. (2021) [76]
Probable lifetime MDD	UKBB [99]	CRP, IL1, IL6, TNFa, ICAM1, P-selectin	The IL6R MR Consortium [104]; CCGC [113] The IL1 Genetics Consortium [114]; de Vries et al. [115]; Paré et al. [116]	2SMR	Cis-SNPs	IL6 → depression (1.38)^e^ CRP → depression (1.18)^e^	Khandaker et al. (2020) [77]
SCZ [103]	PGC	CRP	Ligthart et al. [71]	2SMR	GW-SNPs	CRP **⊣** SCZ (0.91)^a^	Lin et al. (2019) [117]
SCZ [73]	PGC	CRP, IL1Ra, sIL6R	Dehghan et al. [70]; Matteini et al. [118]; Sarwar et al. [105]	2SMR	GW-SNPs; cis SNPs	CRP ⊣ SCZ (0.9)^i^ sIL6R → SCZ (1.06)^i^	Hartwig et al. (2017) [78]
BIP	CGPS-CCHS [74]	CRP	CGPS-CCHS [74]	1SMR	Cis-SNP	n.s.	Wium-Andersen et al. (2016) [119]
ASD [120], BIP [121], MDD [122], SCZ [73]	PGC	CRP	Dehghan et al. [70]	2SMR	GW-SNPs; Cis-SNP	CRP ⊣ SCZ (0.86)^j^	Prins et al. (2016) [123]
SCZ	CGPS-CCHS [74]	CRP	CGPS-CCHS [74]	1SMR	Cis-SNP	n.s.	Wium-Andersen et al. (2014) [74]
Depression	CGPS-CCHS [74]	CRP	CGPS-CCHS [74]	1SMR	Cis-SNP	n.s.	Wium-Andersen et al. (2014)

⊣: Protective effect: increased protein level leads to decreased risk; or increased risk leads to decreased protein level; →: Increased protein level leads to increased risk; or increased risk leads to increased protein level.

*CRP* C-reactive protein, *IL6* interleukin, *(s)IL6R* (soluble) interleukin 6 receptor, *IL6-signaling* proxy IL6 pathway activity by downstream CRP levels, *TNFa* tumor necrotic factor alpha, *IL12* interleukin 12, *IL16* interleukin 16, *IL17* interleukin 17, *IL18* interleukin 18, *IL1RA* interleukin 1 receptor alpha, *IL10* interleukin 10, *IL13* interleukin 13, *IL8* interleukin 8, *MCP1* monocyte chemoattractant protein 1, *sIL2Ra* soluble interleukin 12 receptor alpha, *IL4* interleukin 14, *IL7* interleukin 7, *IL9* interleukin 9, *IL5* interleukin 5, *FGFBasic* basic fibroblast growth factor, *ICAM1* intercellular adhesion molecule 1, *sgp130* soluble glycoprotein 130, *2SMR* two-sample Mendelian randomization, *1SMR* one-sample Mendelian randomization, *LCV* latent causal variable model, *cis-SNPs* selecting instrumental SNPs around the coding genes, *GW-SNPs* selecting genome-wide significant SNPs (5 × 10^−8^), *ADHD* attention-deficiency/hyperactive disorder, *BIP* bipolar disorder, *SCZ* schizophrenia, *MDD* major depression disorder, *PTSD* post-traumatic stress disorder, *AN* anorexia nervosa, *ASD* autism spectrum disorder, *OCD* obsessive-compulsive disorder, *PGC* Psychiatric Genomic Consortium, *UKBB* UK Biobank, *NESDA* the Netherlands Study of Depression and Anxiety study, *CGPS-CCHS* The Copenhagen General Population Study and the Copenhagen City Heart Study.

^a^Odds ratio for per log(mg/l) increase in protein level.

^b^Beta effect for per log(mg/l) increase in protein level.

^c^Not clearly stated by the original paper.

^d^Odds ratio for per unit increase in genetically predicted protein level in log-transformed scale.

^e^Odds ratio per standard deviation increase in genetically predicted protein level.

^f^Beta effect for per log increase in protein level.

^g^Odds ratio per 10–8 g/ml increase in protein levels.

^h^Only from multivariable MR (MVMR).

^i^Odds ratio for two-fold increase in protein level.

^j^Odds ratio for 1 mg/l increases in log(CRP).

Among the studied inflammatory markers, CRP and the IL6 signaling components (IL6, sIL6R, sgp130) are the most studied exposures; other markers have only been examined by few studies. While the statistical models of MR and the strategies for selecting instrumental SNPs varied, CRP was consistently shown to have a protective effect on SCZ. This consistency held even when the CRP GWAS sample size increased from ~80,000 [70] to 200,000 [71] and in an analysis from an independent study [72], and when the size of GWAS for SCZ increased from about 80,000 [73] to 320,000 [52]. The only null finding was from a Danish population cohort with about 78,000 individuals using a one-sample MR design [74]. The protective effect of CRP was also demonstrated for ASD [62] and sub-symptoms of depression [44, 75] but not to major depression disorder (MDD). Another relatively consistent finding is the detrimental effects of increased IL6 signaling to SCZ, MDD, depressive sub-symptoms and depression [44, 75–78]. Significant causal effects for other markers (sIL2Ra and FGFBasic) to psychiatric disorders have been reported by only a single study [79, 80]. In general, there was no causal effects of psychiatric disorders on inflammatory marker levels reported, except by Chen et al. [79] (Table 1). In summary, while sample overlapped, the extremely large sample sizes of the underlying GWAS studies lend some credibility to these causal findings, particularly for CRP and IL6 signaling which have been reported in multiple studies.

Owing to ever increasing samples for GWAS studies, well-powered MR studies are keeping up, discovering novel causal effects of inflammatory markers on psychiatric disorders. However, major challenges remain. Because associations identified by GWAS are typically located in non-coding regions, carefully selecting valid instrumental SNPs is imperative. In the context of studying inflammatory markers as exposures, two popular selection strategies have been used (Table 1): either selecting independent genome-wide significant SNPs (*p* < 5 × 10^−8^) disregarding genomic locations (GW-SNPs), or, among these SNPs, selecting only those that are located to the corresponding coding genes (cis-SNPs). Although the latter seems better in avoiding pleiotropic instruments, these selected cis-SNPs may still be in linkage disequilibrium with SNPs nearby but outside of the coding regions. Another challenge is the highly pleiotropic nature of inflammatory markers, e.g., CRP and IL6 [62]. This phenomenon very likely generates the so-called correlated horizontal pleiotropy which can bias MR estimates (Box 1). To date, most of published studies have not specifically handled this bias [81].

Box 1 Mendelian randomization in cytokine to psychiatric studies

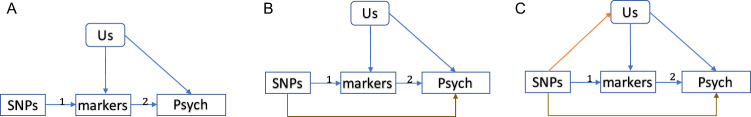

Mendelian randomization (MR) analysis has become a cost-effective design to examine the potential causal effect of immune response to the risk of psychiatric disorders. This design primarily uses recently accumulated large-scale genome-wide association studies (GWAS) data. Despite whether individual-level data (1-sample MR) or GWAS summary statistics are used (2-sample MR), the ideal situation is illustrated in panel A. Here, it is assumed that the effect, *β*_1_, of the genotype (SNPs) on the inflammatory marker (markers) is strong (in MR terminology, the *Relevance* assumption), that there is no relation from genotypes (SNPs) to unmeasured confounder variables (Us) (the *Exchangeability* assumption), and that there is no direct path from the SNPs to the disorders (psych), i.e., the effect of SNPs on the disorders must be mediated by inflammatory markers. In case these assumptions are met, MR analysis is able estimate unbiased causal effects between markers and psychiatric disorders (*β*_2_). Notably, in this ideal situation, in contrast to non-MR models, the causal estimates are less likely being plagued by reverse causation (Psych to markers), nor biased by confounders between markers and psychiatric disorders.

As sample size increases, constructing strong instrument for marker effects can, to a large extent, satisfy the no measurement error assumption (*NOME*), however, to avoid the situation in depicted in panels B and C as difficult. In panel B, genetic instruments may potentially affect the risk of psychiatric disorders which are highly polygenic [124] with effect α. This direct pathway (named horizontal pleiotropy), paralleling to those mediated by inflammatory markers, can bias causal effects estimates. In addition to the direct horizontal pleiotropic pathway, the genetic instruments could potentially also affect unknown confounders (Us), which will allow for indirect horizontal pleiotropic pathway, i.e., from SNPs to Us and to psychiatric disorders, i.e., *γν*. Because these confounders by definition affect the level of inflammatory markers, in this situation it is obvious that the total effect of SNPs (i.e., *β*_1_ + *γη*) on inflammatory markers will correlate with indirect horizontal pleiotropic effect of these SNPs (i.e., γν). This will further challenge to obtain unbiased causal effect estimates.

Standard MR models such as inverse variance weighted model (IVW) [125], egger regression [126], weighted median/mode [127], MR-PRESSO [128], or MR Raps [129] can to certain extent ameliorate the direct horizontal pleiotropic bias but sensibly handling the indirect horizontal pleiotropy is under intensive research recently.

Box 2 Methods Data sources

Data collection was done in November, 2022: we searched PUBMED for articles written in English since January, 2000 that indicated use Mendelian Randomization (MR) study design to study causal relationship between psychiatric disorders and inflammatory biomarkers using the following search terms: (depress* OR schizophrenia OR bipolar OR autism OR attention OR post-traumatic OR anorexia OR psychiatric OR mental) and (inflammat* OR interleukin OR IL-1 OR IL-6 OR C-Reactive OR CRP OR cytokine) and (Mendelian).

Study selection

Two authors (OI/YW) iteratively reviewed titles, then abstracts, before conducting a full-text review of potentially eligible studies. Studies were included if they satisfied all three of the following requirements: [1] use of an MR design, [2] psychiatric disorder as exposure or outcome, and [3] immune system marker as exposure or outcome.

Data extraction

One author (OI) extracted the data and summarized them, while another author (YW) independently examined them. Each MR study’s exposure, outcome, GWAS source, method for MR, instrument selection, findings, sample size, odds ratio (OR) and/or beta (95% CI) were extracted.

Quality assessment

Two reviewers (OI and YW) evaluated the quality of the chosen research; any disparities were discussed, and a third reviewer (EL) settled any differing opinions. The risk of bias in MR studies cannot be assessed using a standardized tool. Therefore, to evaluate the quality of the included studies, we examined how studies approached the selection of valid instruments for MR.

## Discussion and perspectives

We briefly reviewed the evidence of immune dysfunction influence on psychiatric disorders from epidemiological or clinical studies. We added to the growing body of evidence provided by recent genomic and epigenomic findings. We showed that evidence from the fifteen Mendelian randomization studies, which directly tested for causal effects of inflammatory markers on psychiatric disorders, supports a causal interpretation. However, are we able to claim a causal relation between the two?

Synthesizing evidence from these studies leads to several notable points. Epidemiological studies generally support the notion that elevated inflammatory responses may potentially cause psychiatric disorders. The low-level inflammation may stem from either chronic infection history or other illness indexed by sub-clinical but elevated inflammatory marker levels. These data have been most often interpreted as the involvement of the innate immunity arm. However, both genomic and epigenomic studies implied the involvement of adaptive arm as well, even to a large extent reported by a recent study [82] (Figs. 1–3). This is not contradictory to findings from epidemiological studies. The frequently studied inflammatory markers, such as IL6, have well-known functions in modulating the adaptive immune responses, immune cell differentiation, and other cellular processes [83]. The generally small effect sizes in epidemiological and genetic studies indicate that immune dysfunction may be secondary in causing these diseases or it is relevant only for a subset of patients.

Most MR studies have investigated whether perturbations of the IL-6 signaling pathway may lead to increased disease risk. These findings support the epidemiological observations, i.e., increased activity of this pathway plays a causal role in these disorders. But the consistently reported protective effect of CRP is in a startling contrast to prior epidemiological and clinical observations. CRP, an acute-phase protein, responds to acute infections or inflammations by sharply increasing expression levels, up to thousandfold within few hours [84]. But whether common genetic variations identified by GWAS capture these acute response or basal expression levels is uncertain [85–87]. Thus, interpreting elevated CRP levels (<10 mg/l) as indicator of chronic inflammation needs more support: chronic inflammation can lead to elevated CRP levels, but elevated CRP levels may not indicate ongoing chronic inflammation. Moreover, epidemiological studies frequently reported the effects of immune dysfunction within a narrower sampling time window; MR studies, or in general genomic association studies, can only estimate the lifetime average effects. Therefore, this important distinction may lead to divergent conclusions.

The protective effect of CRP is biologically plausible. CRP exists in two isoforms—soluble pentamer and insoluble monomer. In face of infection/inflammation the pentameric form of CRP, mainly produced in the liver, dissociates irreversibly into monomeric form that acts locally and has a pro-inflammatory effect, i.e., by activation of the classical pathway of the complement system [88]. However, CRP-induced complement activation does not lead to the C5-C9 activation, which amplifies pro-inflammatory processes [88]. Thus, the pro-inflammatory effect of CRP is highly regulated and potentially plays a beneficial role for health. In addition, both in vitro and in vivo studies have shown that CRP can opsonize endogenous and exogeneous antigens to facilitate cellular clearance by other immune cells, thereby playing anti-inflammatory and tissue repairing functions [88, 89].

In conclusion, while great progresses have been made in investigating the causal effects of immune dysfunction in psychiatric disorders, we still do not have the full picture. To approach this goal, triangulation of findings from different fields is critical [90]. Currently, most epidemiological or clinical studies have measured inflammatory markers only at baseline in longitudinal studies. As such this design cannot distinguish random fluctuations from true chronic inflammation, which needs multi-timepoints measures of many immune parameters [86, 87]. Although omics technology has brought about novel findings, we are still lacking well-powered cell type specific data to link genetic variations to cellular functions. Lastly, as of today, MR studies are highly biased toward a few, sufficiently sized, datasets, and future work should strive to replicate these findings with independent data sources.
